# Obsessive–compulsive disorder after traumatic injury to the right frontal and left temporal lobes: A case report

**DOI:** 10.1002/pcn5.199

**Published:** 2024-06-14

**Authors:** Daisuke Yoshioka, Takehiko Yamanashi, Teruaki Hayashi, Masaaki Iwata

**Affiliations:** ^1^ Division of Neuropsychiatry Faculty of Medicine, Tottori University Yonago Japan

**Keywords:** cognitive dysfunction, obsessive–compulsive disorder, traumatic brain injury

## Abstract

**Background:**

Obsessive–compulsive disorder (OCD) is a common neuropsychiatric disorder affecting many behaviors in daily life. Hyperactivity of the fronto‐striato‐thalamic circuit via the orbitofrontal cortex (OFC) is assumed to play a major role in the pathophysiology of OCD; however, its pathogenesis is not fully understood. Several reports have described the development of OCD after traumatic brain injury (TBI); however, the pathogenesis of post‐TBI OCD remains unknown. Moreover, patients with TBI often have a variety of sequelae, including cognitive dysfunction and mood disorders, which make the diagnosis and treatment of OCD more complex.

**Case presentation:**

We report the case of a 17‐year‐old Japanese male who developed OCD after traffic trauma. The patient developed a fear of contamination and checking compulsion after injuring his right OFC and left temporal lobe when he ran into a running truck during a suicide attempt. We believe that the patient's fear of contamination can be diagnosed as true post‐TBI OCD. However, his memory impairment was significant, and we considered his checking compulsion to be strongly influenced by cognitive dysfunction due to TBI. We attempted behavioral therapy for OCD; however, sufficient results were not achieved because of the interference from the sequelae of TBI.

**Conclusion:**

It is not rare for OCD symptoms to appear after TBI. Differentiating the OCD symptoms induced by brain injury or cognitive dysfunction associated with TBI is important to determine a treatment strategy.

## Graphical abstract

It is not uncommon for obsessive–compulsive symptoms to appear after traumatic brain injury (TBI). We report the case of a 17‐year‐old Japanese male who developed obsessive–compulsive disorder (OCD) symptoms after TBI due to traffic injury during a suicide attempt. Differentiating whether the symptoms are true OCD induced by trauma or cognitive dysfunction in TBI is important for determining a treatment strategy.

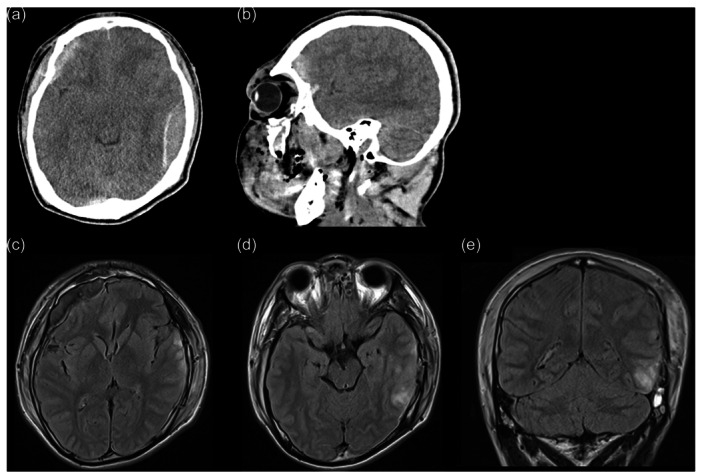

## BACKGROUND

Obsessive–compulsive disorder (OCD) is a common neuropsychiatric disorder that affects many everyday behaviors, such as washing and checking, with a population risk of 2%–3%.^1^ Patients with OCD experience repetitive thoughts, urges, or impulses, causing anxiety and distress, and repeatedly engage in time‐consuming behaviors and mental acts aimed at reducing undesirable obsessions.^2^ Previous studies on idiopathic (nontraumatic) OCD have shown that hyperactivity of the fronto‐striato‐thalamic circuit through the orbitofrontal cortex (OFC) plays a major role in the pathophysiology of OCD.^1^ More recently, the model of this circuit has been modified to include the hippocampus, anterior cingulate gyrus, and basolateral amygdala, which are extensively connected to the OFC, and to ascribe an effective function to this circuit based on current knowledge about the functional importance of these limbic regions and emotional perception.^3^, ^4^ Although these studies examined the general phenotype of OCD, it is possible that there are different brain substrates for different OCD subtypes. Traumatic brain injury (TBI) is a major cause of disability, resulting in common impairments in physical, cognitive, psychological, and behavioral functioning. Behavioral disorders are common in approximately 60% of cases 1 year after trauma, regardless of the initial TBI severity.^5^ The incidence of OCD after TBI is higher than that in the general population, ranging between 1.2% and 30%.^6^, ^7^ One study examined new‐onset OCD symptoms after severe pediatric TBI and reported an incidence rate of approximately 30%.^8^ In general, clinical features of post‐TBI OCD are similar to those of idiopathic OCD.^9^ However, the pathogenesis of post‐TBI OCD remains unclear. For example, cognitive dysfunction, lack of initiative, and rapid mood changes are commonly observed in patients after TBI,^9^, ^10^, ^11^ making the clinical presentation more complex. OCD diagnosis is more challenging and often requires a different treatment approach than idiopathic OCD.

We report the case of a 17‐year‐old Japanese male who developed OCD symptoms after TBI due to traffic injury during a suicide attempt.

## CASE PRESENTATION

**Figure 1:**
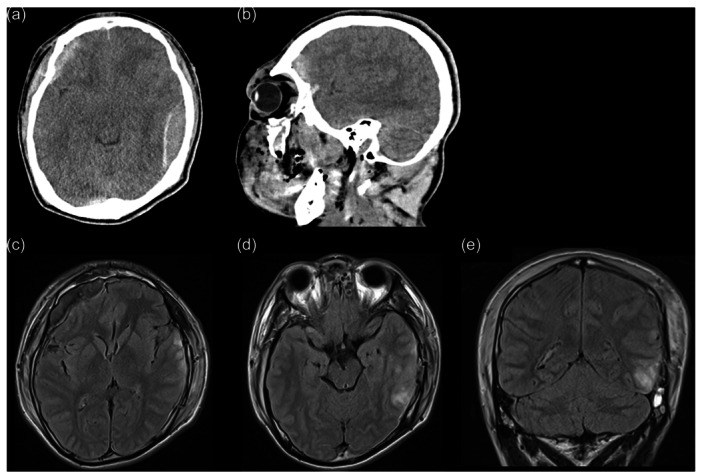
(a,b) Brain computed tomography immediately after head trauma reveals cerebral contusions of the right frontal and left temporal lobes, right frontal subdural hematoma, and left temporal epidural hematoma. (c–e) Fluid‐attenuated inversion recovery sequence brain magnetic resonance imaging 6 days after head trauma shows cerebral contusion of the right frontal and left temporal lobes and right frontal subdural hematoma.

Our patient was a 17‐year‐old right‐handed male high‐school student. His parents had divorced when he was 2 years old, and since then he had lived with his mother, grandmother, and older brother. He had a medical history of irritable bowel syndrome (IBS) and had needed to use the bathroom several times a day since elementary school. He had always had a bright personality and enjoyed training at the athletics club after entering high school. However, in the fall of his 16th year, without inducement, he began to suffer more from IBS symptoms, became indifferent to socializing with his friends, and gradually lost the will to live. In the spring of his 17th year, he ran into a running truck durinig a suicide attempt and was injured. He was immediately taken to hospital, where he was diagnosed with cerebral contusions of the right frontal and left temporal lobes, right frontal subdural hematoma, left temporal epidural hematoma, multiple skull fractures, and tension pneumothorax (Figure 1). Emergency surgery was performed. He had appeared normal to his family just before the accident, and the patient had no memory of several months before or 1 week after the accident; thus, the exact reason for his suicide attempt was unclear. After weaning from the ventilator on the 5th day after the accident, the patient quickly regained consciousness, and his psychiatric condition remained stable. The Wechsler Adult Intelligence Scale was used 3 weeks after the accident to assess the effects of the brain injury, and his full‐scale IQ was 78 (Verbal Comprehension Index, 83; Perceptual Reasoning Index, 84; Working Memory Index, 85; and Processing Speed Index, 75). The cognitive impairment due to the accident was not significant compared to his life before the head injury. One month after the accident, the patient was diagnosed with depression with the reappearance of suicidal thoughts. Because he had conducted a serious suicide attempt, he was prescribed brexpiprazole (0.5 mg) first, rather than standard antidepressants, such as selective serotonin reuptake inhibitors (SSRIs). His depressed mood improved quickly; however, he began to feel that his surroundings were filthy and frequently washed his hands. He spent more time in the bathroom and repeatedly washed his buttocks. He also began to refuse food for fear of using the bathroom. In addition, his depressive mood recurred. He was diagnosed with OCD and prescribed fluvoxamine (150 mg) for the obsessive–compulsive and depressive symptoms; however, his symptoms did not improve. Three and a half months after the accident, he was admitted to our hospital for further treatment.

**Figure 2:**
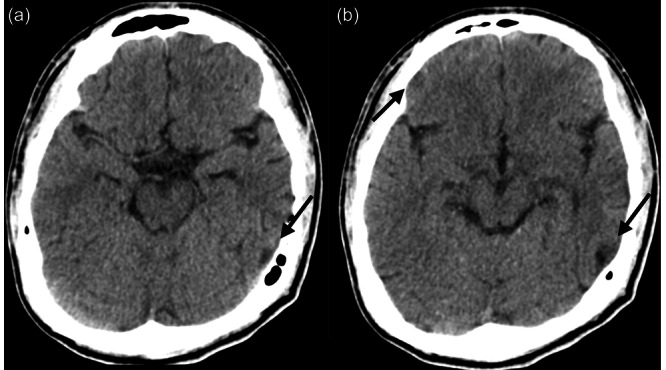
Brain computed tomography 5 months after the accident reveals known cerebral contusions in the right orbitofrontal cortex and left temporal lobe.

In the hospital ward, because of the fear of contamination, he avoided touching anything and spent hours washing his hands. The Yale–Brown Obsessive–Compulsive Scale (Y‐BOCS) score was 29. Before initiating exposure and response prevention (ERP) treatment for OCD, we explained to him the pathophysiology of OCD. The patient's comprehension and insight were poor, and he understood the concept of treatment after receiving repeated explanations. However, the patient frequently forgot the explanation, and lacked awareness of the irrationality of obsessive thoughts. After ERP was started, his fear of contamination reduced, he was able to touch anything, and his depressive mood disappeared accordingly. However, his mood fluctuated easily in the absence of triggers, and he abandoned the treatment, following which his OCD symptoms worsened each time. In addition, the patient developed another compulsion to check his smartphone repeatedly, fearing that he might have misused it and lost data. OCD and affective symptoms existed independently but interacted with each other; when one worsened, the other worsened as well. To reconsider the patient's condition, computed tomography of the head was performed 5 months after the accident, which revealed known cerebral contusions in the right OFC and left temporal lobe (Figure 2). Additionally, the Wechsler Memory Scale‐Revised revealed significant memory impairment in verbal memory (68), visual memory (<50), general memory (<50), attention (81), and delayed memory (72), which was suspected to be related to poor comprehension and OCD symptoms, especially checking compulsions. Two months after admission, he requested treatment discontinuation and was discharged from the hospital, although he was still undergoing treatment. The final Y‐BOCS score was 24, which was not a satisfactory treatment outcome.

## DISCUSSION

Several cases of OCD following TBI have been reported.^12^, ^13^, ^14^ However, the diagnosis of OCD in the context of TBI is complicated because of the potential for overlapping symptomatology between the conditions.^15^ Cognitive dysfunction associated with TBI may mimic clinical manifestations of idiopathic OCD. For example, memory deficits following TBI may lead to excessive task checking, as has been observed in patients with OCD. Therefore, it is difficult to determine whether a particular symptom reflects the well‐known cognitive dysfunction characteristics of TBI or new‐onset OCD. After TBI, it is often necessary to leave a familiar life and adapt to a new environment. This may lead to excessive checking, repetitive behaviors, and anxiety associated with these behaviors, which can be mistaken for new‐onset OCD caused by TBI. It should also be emphasized that there is limited research supporting the pathophysiological relationship between damage to specific brain lesions or pathways following TBI and subsequent OCD.

In the present case, cognitive dysfunction, particularly memory dysfunction, was observed due to TBI. The patient's symptoms of repeatedly checking the smartphone for mishandled data may have been due to compensatory actions against the memory impairment caused by damage to the temporal lobe. In such cases, rehabilitation for cognitive dysfunction rather than the standard OCD treatment is needed. An accurate diagnosis of OCD following TBI determines an appropriate treatment strategy and thus requires a careful approach, including neuropsychological testing.^15^

In contrast, excessive washing, in this case, was related to the fear of contamination and cannot be interpreted as a compensatory behavior for cognitive dysfunction or as a coping behavior for anxiety following TBI and could be diagnosed as true post‐TBI OCD. A study investigating the predictors of new‐onset obsessive–compulsive symptoms after TBI reported associations with female sex, psychosocial adversity, and prefrontal/temporal lesions but no association with injury severity.^8^ Notably, OCD may occur soon or several months after TBI, in most cases, after a patient experiences a major depressive episode.^16^, ^17^ In our case, we cannot conclude that the patient had psychological problems at the time of the accident because he had no memories before or after the traffic accident, and his behavior appeared normal to the family before the trauma. However, the patient was distressed by the IBS symptoms and interpersonal relationships, resulting in a suicide attempt. Furthermore, a high comorbidity of IBS and OCD has been reported,^18^, ^19^ and it has been hypothesized that IBS is considered “bowel obsessional syndrome” and that there is a common pathology between the two.^20^ The episodes of frequent bathroom use in a day, observed since elementary school, could be interpreted as though the patient had already had OCD symptoms since then. These factors may play a role in the development of post‐TBI OCD. Further, it is consistent with a typical case in which our patient became depressed after TBI and subsequently developed OCD. The dysfunction of neurotransmitter systems, including serotonin and dopamine, has been reported in TBI patients^21^ and may be involved in the development of depression and subsequent OCD.^22^ In this case, brexpiprazole was partially effective against depression, suggesting neurotransmitter system dysfunction.

A report on acquired brain lesions indicated that injuries in several brain regions were associated with the onset of OCD symptoms and that damage to the cortical‐striatal‐pallidal‐thalamo‐cortical circuit, specifically the frontal lobes and basal ganglia, appeared to be intrinsically related to OCD symptoms.^23^ There have also been some reports of checking and repetition compulsions, as observed in this case, after temporal lobe lesions, which may have been the result of impaired memory formation in the temporal cortex.^24^, ^25^ Our patient had damage to the right OFC and the left temporal lobe. Multiple studies indicate that the OFC is involved in representations of reward and punishment,^26^ anxiety and emotion processing,^27^ and inhibitory control.^28^ Obsessions are classically assumed to reduce anxiety and suggest underlying inhibitory deficits.^29^ It is reasonable to hypothesize that the OFC lesions, in this case, played an important role in OCD development.

Despite increasing interest in post‐TBI OCD, no evidence‐based guidelines or recommended specific treatments are available; however, standard treatments for idiopathic OCD, such as SSRI‐based pharmacotherapy, psychotherapy, and electroconvulsive therapy, are used.^30^ In our case, the patient was treated with a combination of pharmacotherapy with fluvoxamine and ERP; however, only a minor improvement in the obsessive–compulsive symptoms was achieved. The reasons for this may be poor insight, resulting in an insufficient understanding of the treatment strategy, and lack of initiative or easily fluctuating mood, preventing continuous treatment. Poor insight, lack of motivation for treatment, and depression can interfere with OCD treatment and have been reported to correlate with OCD severity.^31^, ^32^, ^33^ In the first place, the dropout rate of ERP for OCD is as high as 25%,^34^ and it is assumed that patients with post‐TBI OCD are more likely to experience treatment failure. In the present case, a more attentive approach to mood and motivation and more careful neuropsychoeducation for post‐TBI sequelae might have allowed the patient to continue treatment and achieve greater improvement in OCD symptoms.

## CONCLUSION

It is not uncommon for obsessive–compulsive symptoms to appear after TBI. However, OCD diagnosis in the context of TBI is complicated by the potential for overlapping symptoms between the two conditions. Determining whether the symptoms are true OCD induced by trauma or cognitive dysfunction in TBI is important for choosing a treatment strategy. Even when a diagnosis of post‐TBI OCD is made, consideration of both the OCD symptoms and the sequelae of TBI may determine the success or failure of OCD treatment.

## AUTHOR CONTRIBUTIONS

**Daisuke Yoshioka** treated the patient and drafted the manuscript. **Takehiko Yamanashi**, **Teruaki Hayashi** and **Masaaki Iwata** critically reviewed the draft and revised it. All authors approved the final version of the manuscript.

## CONFLICT OF INTEREST STATEMENT

The authors declare no conflict of interest.

## ETHICS APPROVAL STATEMENT

This study was conducted according to the principles of the Declaration of Helsinki.

## PATIENT CONSENT STATEMENT

Informed written consent and a signed release were obtained from the patient for the publication of this report and any accompanying images.

## CLINICAL TRIAL REGISTRATION

Not applicable.

## Data Availability

Data sharing is not applicable to this article.
